# Enantioselective Utilization of D-Amino Acids by Deep-Sea Microorganisms

**DOI:** 10.3389/fmicb.2016.00511

**Published:** 2016-04-19

**Authors:** Takaaki Kubota, Tohru Kobayashi, Takuro Nunoura, Fumito Maruyama, Shigeru Deguchi

**Affiliations:** ^1^Research and Development Center for Marine Biosciences, Japan Agency for Marine-Earth Science and TechnologyYokosuka, Japan; ^2^Department of Microbiology, Graduate School of Medicine, Kyoto UniversityKyoto, Japan

**Keywords:** D-amino acid deaminase, D-amino acid utilization, D-valine, deep sea, marine bacteria

## Abstract

Microorganisms that utilize various D-amino acids (DAAs) were successfully isolated from deep-sea sediments. The isolates were phylogenetically assigned to Alphaproteobacteria, Gammmaproteobacteria, and Bacilli. Some of the isolates exhibited high enantioselective degradation activities to various DAAs. In particular, the Alphaproteobacteria *Nautella* sp. strain A04V exhibited robust growth in minimal medium supplemented with D-Val as a sole carbon and nitrogen source, whereas its growth was poor on minimal medium supplemented with L-Val instead of D-Val. Its growth was facilitated most when racemic mixtures of valine were used. In contrast, the *Nautella* strains isolated from shallow-sea grew only with L-Val. No significant differences were found among the strains in the genome sequences including genes possibly related to DAA metabolisms.

## Introduction

Homochirality is one of the most important features of biological systems. Why and how life on earth chose L-amino acids (LAAs) instead of D-amino acids (DAAs) to build proteins remains open questions related to the chemical origins of life (Siegel, 1998; Blackmond, 2010; Hein and Blackmond, 2012). In the context of the homochirality of life, it was considered that organisms preferentially utilized LAAs over DAAs. However, recently, there have been a number of reports that the organisms utilize DAAs.

It is well-known that the peptidoglycan in bacterial cell walls comprise of DAAs (Schleifer and Kandler, 1972; Vollmer et al., 2008; Typas et al., 2011). The glycan strands are made of *N*-acetylglucosamine and *N*-acetylmuramic acid units that are connected by β-1,4-glycosidic linkages, and short peptide chains that cross-link the strands are enriched with D-Ala and D-Glu. Other DAAs such as D-Met, D-Val, D-Phe, and D-Trp are also found in the peptidoglycan (Lam et al., 2009; Cava et al., 2010). DAAs are also found in various antibiotic peptides that are produced by bacteria (Brückner and Fujii, 2010a,b; Velkov et al., 2010; Tempelaars et al., 2011; Doveston et al., 2012).

DAAs even mediate vital physiological functions in mammals. In the central nerve system, for example, D-Ser that is racemized from L-Ser by serine racemase acts as a co-agonist for the *N*-methyl-D-aspartate receptor and controls synaptic plasticity and memory functions (Baumgart and Rodríguez-Crespo, 2008; Billard, 2012). Irukayama-Tomobe et al. (2009) reported that aromatic DAAs (D-Phe and D-Trp) act as bacteria-derived chemoattractants that elicit a chemotactic response in human neutrophils. They also suggested that D-Phe and D-Trp had lipid-lowering effects in human blood.

Identification of metabolic pathways relating to DAAs is crucial to understand their physiological functions. Such knowledge may provide novel insights into various pathophysiological conditions, thereby leading to development of new therapeutic strategies. However, our knowledge of the diversity of DAA metabolic pathways is still extremely limited.

The rates of DAA utilization and degradation are significantly lower than those of LAAs in soil and oceanic waters (Hopkins and Ferguson, 1994a; Hopkins et al., 1994b; O'Dowd et al., 1999; Amon et al., 2001; Calleja et al., 2013; Azúa et al., 2014). DAAs have been detected in diverse environments such as soil (Pollock et al., 1977), rivers (Stepanauskas et al., 2000; Dittmar et al., 2001; Tremblay and Benner, 2009), lakes (Kawasaki and Benner, 2006), and oceans (Lee and Bada, 1977; McCarthy et al., 1998; Dittmar et al., 2001; Kawasaki and Benner, 2006; Wedyan and Preston, 2008). Intriguingly, the abundance of DAAs among the total amino acids was higher in oceanic dissolved organic matter (DOM) than in terrestrial DOM (Dittmar et al., 2001; Kawasaki and Benner, 2006).

In oceanic environments, the presence of DAA-utilizing microbes was suggested by the activity measurements. In deep-sea water, the greater assimilation rate of D-Asp than that of L-Asp was observed (Perez et al., 2003). However, microbes that utilize DAAs as carbon and nitrogen sources have not been previously isolated from marine environments. Here, we report the first isolation of DAA-utilizing bacteria from deep-sea sediments. Characteristics of physiological and genomic traits of a representative strain are also given.

## Materials and methods

### Strains

*Nautella italica* LMG24365 was provided by the Belgian Co-ordinated Collections of Micro-organisms/Laboratory of Microbiology UGent (BCCM/LMG) Bacteria Collection (Ghent, Belgium). *N*. *italica* strain R11 was kindly provided by Dr. Torsten Thomas (University of New South Wales, Sydney, Australia).

### Screening of DAA-utilizing microorganisms

Deep-sea sediments were collected from Sagami Bay by manned submersibles, *Shinkai* 2000 and *Shinkai* 6500, and by a remotely operated vehicle, *ROV Hyper Dolphin*, during Japan Agency for Marine-Earth Science and Technology (JAMSTEC) expeditions (Cruise NT01-11, NT04-06, YK05-15, NT06-01, NT06-17, YK07-05, NT08-24) (Table S1). The samples were stored at −80°C.

To enrich DAA-utilizing bacteria in the sediments, two types of media, DBM and DAM, were prepared using artificial seawater (Daigo's artificial seawater SP for marine microalgae medium, Nihon Pharmaceutical Co. Ltd., Tokyo, Japan) as a basal medium. DBM was used for screening of bacteria utilizing branched-chain DAA, and composed of artificial seawater (pH 8.0) supplemented with 0.005% (wt/v) D-valine, 0.005% D-leucine, 0.1% glucose, 0.001% yeast extract (Becton Dickinson, Franklin Lakes, New Jersey, USA), and 0.005% K_2_HPO_4_. DAM was used for screening of bacteria utilizing aromatic DAAs, and composed of artificial seawater (pH 8.0) supplemented with 0.005% D-phenylalanine, 0.005% D-tryptophan, 0.1% glucose, 0.001% yeast extract, and 0.005% K_2_HPO_4_. DAAs and LAAs were obtained from Wako Pure Chemical Industries (Osaka, Japan). When the media was prepared for isolating colonies on plate, 1.5% (wt/v) agar was added to DBM and DAM.

Slurry of the deep-sea sediments was inoculated into 2 mL of DBM or DAM in 5-mL test tubes. The cultures were incubated at 30°C for 5 days with shaking at 130 rpm. Afterwards, 0.02 mL of enrichment was inoculated into fresh medium, and incubated again under the same conditions. This procedure was repeated three times. Each enrichment cultures thus obtained were spread onto glucose-deficient DBM or DAM plates and incubated at 30°C. Morphologically distinct colonies were selected, transferred into 2 ml of the same liquid medium, and incubated at 30°C with shaking at 130 rpm. The resulting cultures were then spread onto solid media and incubated at 30°C. This purification procedure was repeated twice. The colonies in this secondary enrichment culture were isolated as DAA-utilizing bacteria.

### Nucleotide sequences of 16s rRNA genes and phylogenetic analysis

A universal primer set of bacterial 16S rRNA gene was used for PCR amplification of the 16S rRNA genes from the isolates (Lane, 1991). A colony of each isolate was used as a template in the PCR reaction with LA Taq DNA polymerase (Takara Bio, Otsu, Japan) using a thermal cycler (model ABI 9700; Applied Biosystems, Foster City, CA, USA). The PCR products were directly sequenced using a DNA sequencer (ABI3130; Applied Biosystems) and an ABI Prism BigDye terminator sequencing kit (Applied Biosystems). 16S rRNA gene sequences are deposited in DDBJ/EMBL/GenBank database under accession numbers summarized in Table S2.

### Preparation of resting-cell suspensions

*N. italica* LMG24365, *N. italica* strain R11, and our isolates were pre-cultured in 20-mL test tubes containing 4 mL Difco Marine Broth 2216 (MB; Becton Dickinson) at 30°C with shaking at 120 rpm. Aliquots (0.5 mL) of the pre-culture were transferred to 50 mL MB in 200-mL Erlenmeyer flasks and incubated at 30°C with shaking at 180 rpm. Cells were harvested during the pre-stationary phase by centrifugation at 10,000 × g for 15 min at 4°C, washed twice with 3% NaCl, and once with 50 mM HEPES-NaOH buffer (pH 7.5). The washed cells were suspended in 50 mM HEPES-NaOH buffer (pH 7.5) containing 10% dimethyl sulfoxide. The cell concentration was adjusted to 100 mg wet cell mL^−1^ and stored at −80°C.

Before activity measurements, the cell concentrations of the resting cell samples were measured turbidimetrically at 660 nm (OD_660_) after diluting the culture broths with the appropriate medium. The optical density value was converted into the dry cell weight (DCW) using a calibration equation that was established by plotting the weight of a known volume of desiccated cells. The desiccated cells were prepared by washing the wet-cell samples (usually from 45 mL culture broth) twice with pure water and drying them at 105°C for 12 h.

### Assay of amino acid degradation activity

The amino acid degradation activity was measured based on the quantity of α-keto acid produced from each amino acid (AA) using the 3-methyl-2-benzothiazolinone hydrazone (MBTH) method (Soda, 1968). Various DAAs and LAAs (aliphatic AAs: Ala, Val, Leu, and Ile; aromatic AAs: Phe, and Trp; hydroxyl AAs: Ser and Thr; sulfur-containing AAs: Cys and Met; AA amides: Asn and Gln; imino acid: Pro; acidic AAs: Asp and Glu; and basic AAs: Lys, Arg, and His) were used as substrates.

The reaction mixture (50 μL) was composed of 50 mM HEPES-NaOH buffer (pH 7.5), 50 mM DAA or LAA, and 0.5–6.75 μg-DCW mL^−1^ resting cells. The reaction was performed at 30°C for 60 min and terminated by heating at 96°C for 5 min. The reaction mixture was centrifuged at 10,000 × g at room temperature for 15 min to separate the cells, and 20 μL of supernatant was taken and mixed with 10 μL 1 M sodium acetate (pH 5.0) and 10 μL 25 mM MBTH. The mixture was incubated at 50°C for 30 min and then cooled to room temperature. The absorbance was measured at 320 nm against a blank, which contained all of the components except amino acids. α-Ketoglutaric acid was used as a calibration standard.

For determination of the kinetic parameters, the reaction was performed at 30°C for 30 min in 50 mM HEPES-NaOH buffer (pH 7.5) with 1, 5, 25, 50, 100, or 125 mM substrate, and 0.5–6.75 μg-DCW mL^−1^ resting-cells.

### D- and L-valine degradation studies

*Nautella* sp. strain A04V and *N. italica* LMG24365 were pre-cultured in 20-mL test tubes containing 5 mL MB for 12–15 h at 30°C with shaking at 120 rpm. Cells were harvested by centrifugation at 5,000 × g for 5 min, and washed three times with sterile minimal medium composed of 2.6% (wt/v) NaCl, 0.08% KCl, 0.56% MgCl_2_·6H_2_O, 0.76% MgSO_4_·7H_2_O, 0.00005% FeSO_4_, 0.154% CaCl_2_·2H_2_O, and 0.01% Na_2_HPO_4_ (pH 7.8). Cells were suspended in the same medium and the cell density was adjusted to OD_660_ = 0.1. Aliquots (0.05 mL) of the cell suspension were inoculated into 5 mL minimal medium in L-shaped test tubes, which contained 6 mM D-, and L-valine mixtures (D:L = 0:6, 1:5, 2:4, 3:3, 4:2, 5:1, or 6:0) as the sole carbon and nitrogen source. For each strain, seven cultures were prepared in duplicate and incubated at 30°C for 250 h with shaking at 70 rpm. The OD_660_ was recorded automatically every 30 min using an Advantec TVS062CA biophotorecorder (Advantec Toyo Co. Ltd., Tokyo, Japan). The morphologies of the cultured cells were observed using an Olympus BX51 microscope (Tokyo, Japan).

### Analysis of L- and D-valine using HPLC

The valine concentration was measured using a Shimadzu HPLC Prominence system (Shimadzu Corporation, Kyoto, Japan). Valine was derivatized to a fluorescent diastereomer, and detected by using a Shimadzu fluorimeter (RF 20Axs, Kyoto, Japan). The excitation and emission wavelengths for detection were set to 350 and 450 nm, respectively.

A 30 μL aliquot of the culture medium was sampled during each growth phase of the cultures, and centrifuged at 10,000 × g at 4°C for 15 min. The supernatant was diluted to the appropriate concentrations with distilled water. A 10 μL aliquot was then mixed with 10 μL 0.1 N HCl, 80 μL 0.2 M borate-NaOH buffer (pH 10) containing 1.75 mg mL^−1^ *N*-isobutyryl-L-cysteine, and 1.25 mg mL^−1^ *o*-phthalaldehyde. The mixture was filtered through 0.20 μm PTFE cartridge filters (Millipore, MA, USA) and then injected into an RP-HPLC column (YMC-pack Pro C18, 4.6 mM i.d. × 250 mM). Gradient elution was applied using the following eluents: A, 10 mM sodium-borate/10 mM sodium-phosphate buffer (pH 8.2); B, 40% methanol/40% acetonitrile/10% water. The gradient program was as follows: 35% B for 1 min; 35–60% B for 17 min; and 60% B for 3 min. The derivatized pure L- and D-valine were used as calibration standards.

### Draft genome sequencing, mapping, assembly, and annotation

Genomic DNA was extracted from strains A04V and LMG24365 using Qiagen DNeasy Tissue Kits (Qiagen, Valencia, CA, USA). Genomic DNA sequencing of the strains was performed at the Beijing Genome Institute (BGI; Shenjun, China) using the Illumina HiSeq 2000 platform (Illumina Inc., San Diego, CA, USA) with the single-end strategy for 90-bp reads. The quality of the reads was checked and trimmed (Phred quality score >15) with FASTQ tools using the online data analysis platform Galaxy (Goecks et al., 2010). The sequencing reads were aligned with the *N*. *italica* strain R11 genome reference using Bowtie2 software version 2.1.0 with the default settings. The unmapped reads of strain A04V in the reference genome were assembled into 2587 contigs using ABySS software version 1.3.2 (using kmer = 31). The protein-coding sequences (CDSs) were identified using GLIMMER software version 3.0.2 (Delcher et al., 2007) and those shorter than 50 codons were eliminated. Functional assignments of the predicted genes were made using BLASTP (*e* ≤ e−5) with the GenBank and Pfam databases (pfam-A, version 26.0; Punta et al., 2012). Draft genome sequences are deposited in DDBJ/EMBL/GenBank database under PRJDB4276 and PRJDB4510 (Table S2).

## Results and discussion

### Enrichment and isolation of DAA-utilizing bacteria from deep-sea sediments

To isolate DAA-utilizing bacteria, we used branched-chain DAAs (D-Val and D-Leu) and aromatic DAAs (D-Phe and D-Trp) as the substrates for enrichment. These DAAs have been detected as minor D-forms in the Sargasso Sea (D-Ile) (Lee and Bada, 1977), in the Equatorial Pacific (D-Leu) (Lee and Bada, 1977; McCarthy et al., 1998), and in the Gulf of Mexico (D-Phe) (McCarthy et al., 1998).

Fifty six sediment samples from Sagami Bay, Japan (at water depths ranging from 800 to 1,500 m, Table S1) were inoculated into DBM and DAM, and incubated aerobically to obtain enrichment cultures of DAA-degrading bacteria using a total of 112 tubes. After enrichment and subsequent isolation of colonies, we obtained 17 and 11 isolates that produced morphologically distinct colonies on DBM and DAM agar plates, respectively. The purity of the isolates was confirmed by microscopic observation and by sequencing their 16S rRNA genes.

**Figure 1:**
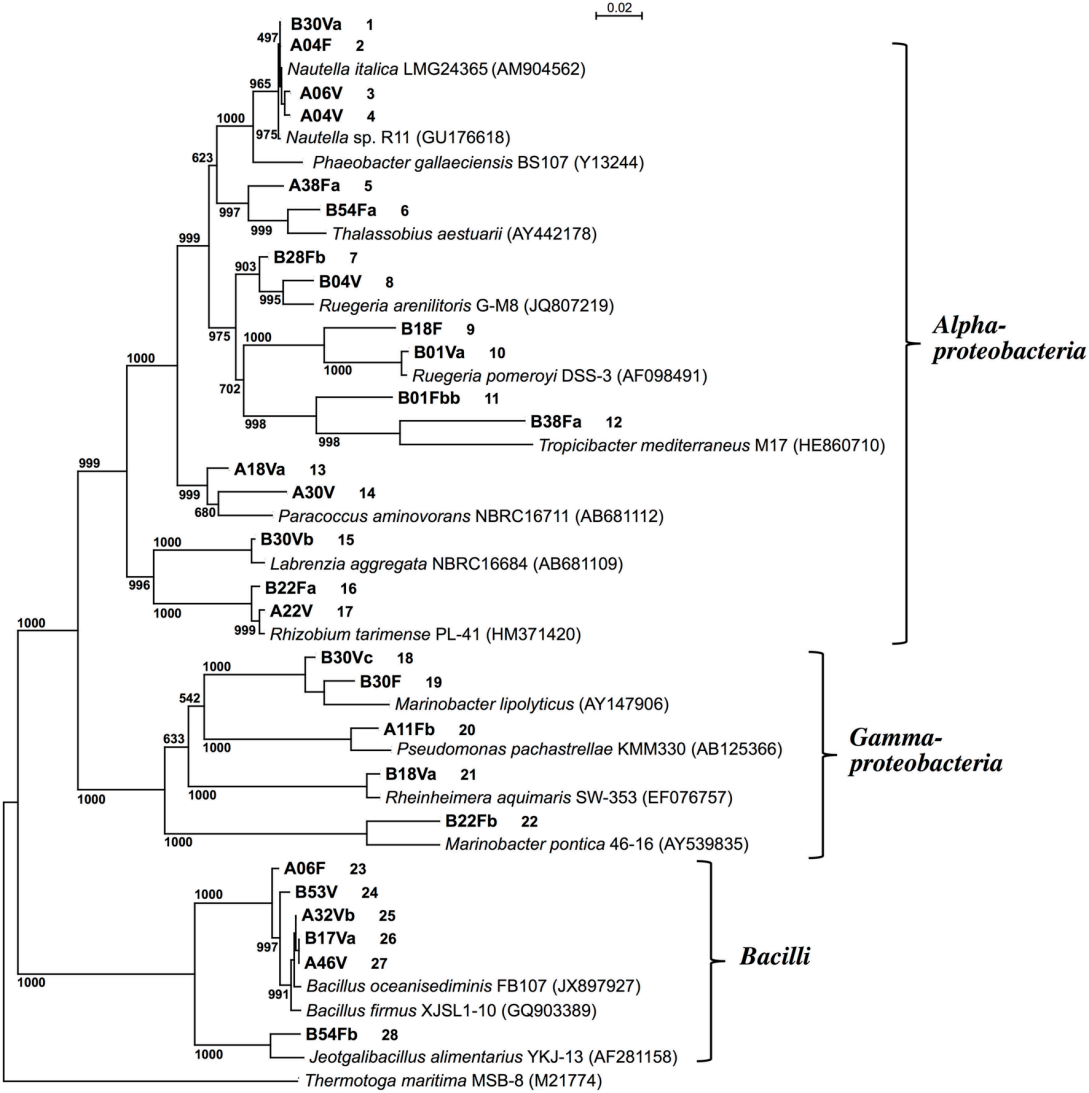
**Phylogenetic tree based on the 16S rRNA gene sequences of D-amino acid-degrading isolates from deep-sea sediments**. *Thermotoga maritima* MSB-8 was used as an outgroup. The phylogenetic tree was constructed using the neighbor-joining method (Saitou and Nei, 1987). The accession numbers of the sequences are shown in parentheses. Numbers at each node indicate the bootstrap value based on 1000 replicates. Strains obtained in this study are shown in bold and the first letter of the strain number indicates the screening medium used: DAM (A) or DBM (B). Figures following the strain numbers are identifiers employed in this study.

Partial nucleotide sequences (0.8–1.4 kb) of the 16S rRNA genes of the 28 isolates showed that 17 strains belonged to Alphaproteobacteria, 5 strains belonged to Gammaproteobacteria, and 6 strains belonged to Bacilli (Figure 1). There were no significant relationships between the taxonomic diversity of the DAA-degrading bacteria and the two types of screening media used for their enrichment and isolation.

### Determination of the DAA degradation activities of the isolates

**Figure 2:**
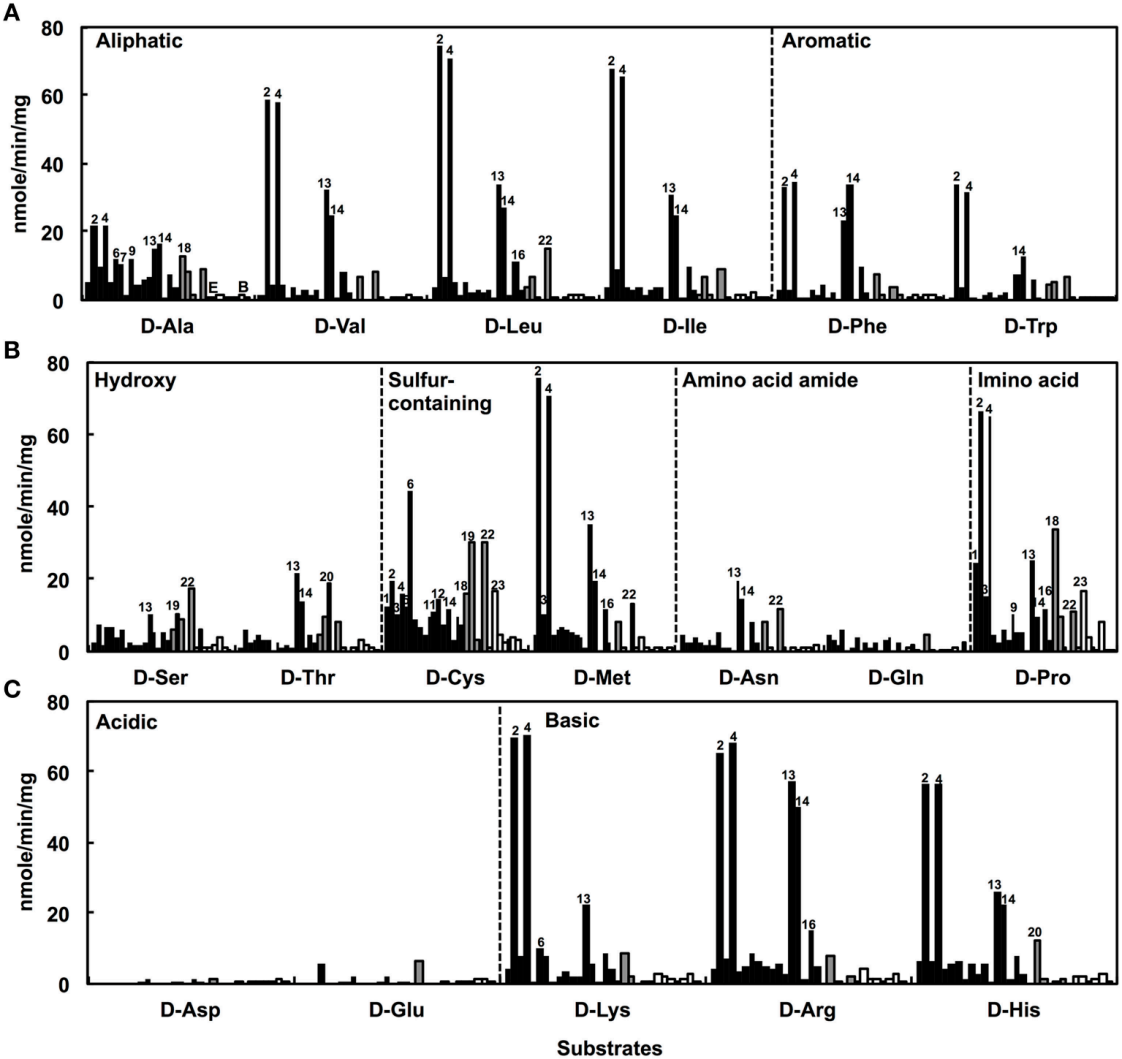
**Deamination of D-amino acids to α-keto acids using the resting cells of strains isolated from deep-sea sediments as catalysts**. Neutral D-amino acids **(A,B)** and ionic D-amino acids **(C)** were used as substrates. Reactions were performed at 30°C for 1 h. Bars indicate the degradation activities of each isolate. Black, gray, and white colors indicate phylogenetic affiliations, i.e., Alphaproteobacteria, Gammaproteobacteria, and Bacilli, respectively. Numbers indicate the identifiers of isolates as shown in Figure 1.

**Figure 3:**
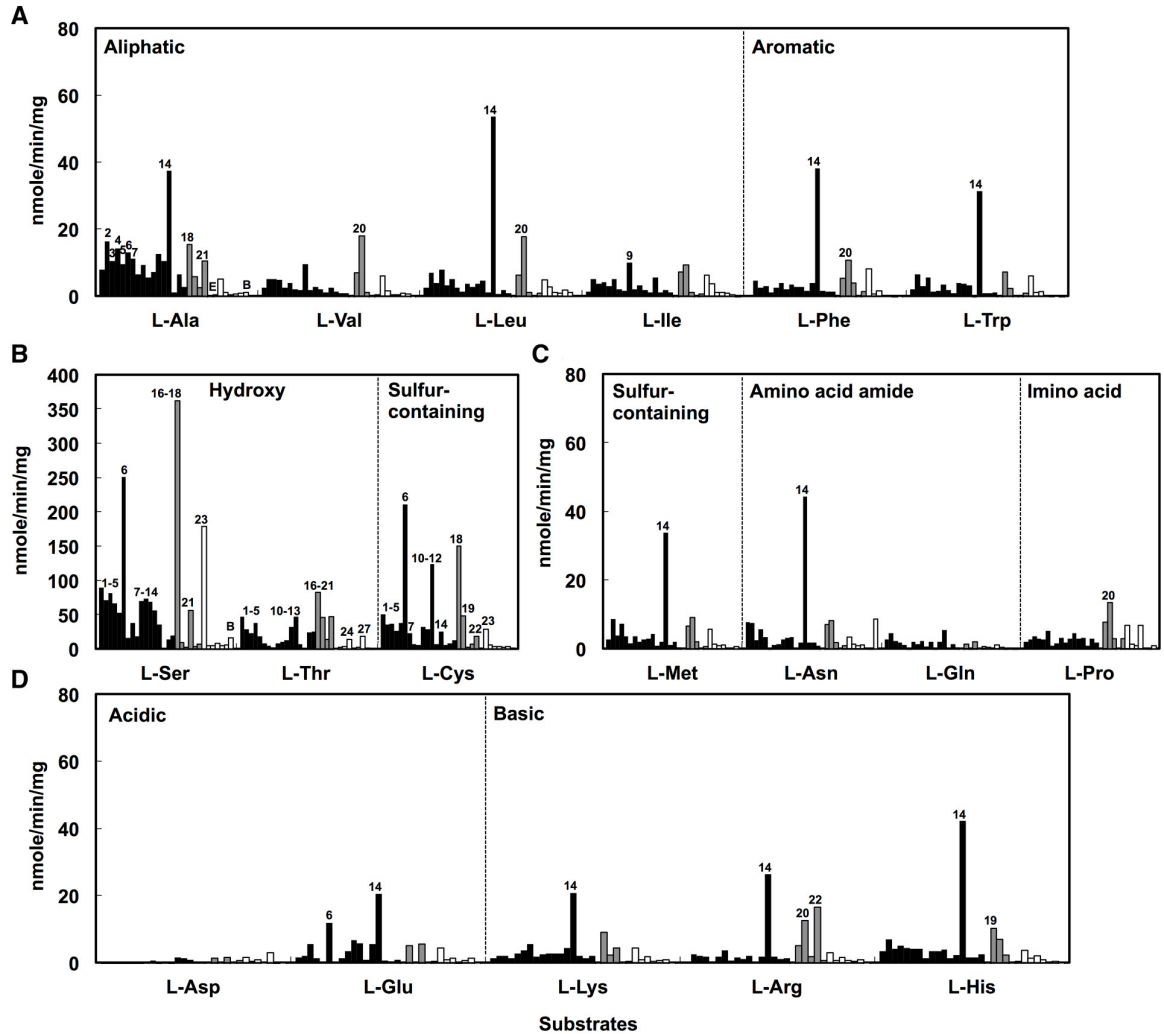
**Deamination of L-amino acids to α-keto acids using the resting cells of D-amino acid-degrading strains isolated from deep-sea sediments as catalysts**. Neutral L-amino acids **(A–C)** and ionic L-amino acids **(D)** were used as substrates. Reactions were performed at 30°C for 1 h. Bars indicate the degradation activities of each isolate. Black, gray, and white colors represent phylogenetic affiliations, i.e., Alphaproteobacteria, Gammaproteobacteria, and Bacilli, respectively. Numbers indicate the identifiers of isolates as shown in Figure 1.

The DAA degradation activities of the isolates were examined using a spectrometric method with resting cells (Figure 2). The alphaproteobacterial strains, i.e., *Nautella* sp. strain A04V and A04F (numbers 2 and 4 in Figure 2, respectively), and *Paracoccus* sp. strain A18Fa and A30V (numbers 13 and 14 in Figure 2, respectively), showed high DAA degradation activities compared with the others. Interestingly, the strains A04V, A04F, and A18Fa degraded various DAAs, but in most cases, did not degrade their L-counterparts in this reaction condition (Figure 3). The resting cells of the alphaproteobacterial isolates degraded not only the branched-chain and aromatic DAAs that were supplemented to the culture media for enrichment and isolation, but also D-Met and D-Pro as well as basic DAAs, i.e., D-Lys, D-Arg, and D-His. The isolates with no or weak DAA degradation activities showed poor growth on the glucose-deficient DBM or DAM. We also evaluated the amino acid racemase activities in resting cells according to the method described by Asano and Endo (1988), but did not observe significant activity except for alanine.

The presence of metabolic pathways to utilize DAAs has been reported in Gammaproteobacteria, i.e., *Pseudomonas fluorescens* (Tsukada, 1966), *Pseudomonas aeruginosa* (Marshall and Sokatch, 1968), *Salmonella typhimurium* (Wild et al., 1974), and *Escherichia coli* (Raunio et al., 1973); Epsilonproteobacteria, i.e., *Helicobacter pylori* (Tanigawa et al., 2010); and Bacilli, i.e., *Ureibacillus thermosphaericus* (Akita et al., 2014). *P. aeruginosa* PAO1 is known to utilize six DAAs (D-Arg, D-Glu, D-Gln, D-Ala, D-Asn, and D-Val) as the sole carbon and nitrogen source in bacterial cultures, and harbor two amino acid dehydrogenases (LAA dehydrogenase and DAA dehydrogenase) for utilization of DAAs (Li et al., 2010). However, it remained unclear whether the pathway of DAA utilization is ubiquitously present among marine bacteria (Zhang et al., 2016). Our results suggest the presence of metabolic pathways to utilize DAAs in marine Alphaproteobacteria.

### Growth behavior of *nautella* sp. strain A04V

Among the four strains of *Nautella* that exhibited degradation activities to DAAs (Figure 2), strain A04V had the broadest DAA substrate range, i.e., D-Ala, D-Val, D-Leu, D-Ile, D-Asp, D-Glu, and D-Phe. This strain grew on these DAAs and also utilized D-Ser, D-Thr, D-Cys, or D-Arg as the sole carbon and nitrogen source on solid media. Therefore, we used strain A04V to characterize marine DAA-degrading bacteria in greater detail. *Nautella* strain A04V was closely related to *N*. *italica* strain LMG24365 and strain R11 based on 16S rRNA gene similarity analysis (100% similarity).

**Figure 4:**
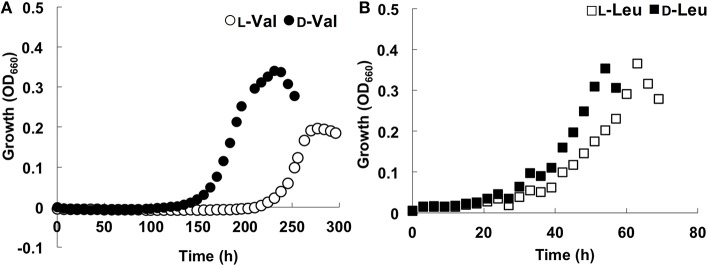
**Growth curves of ***Nautella*** sp. strain A04V**. **(A)** In the presence of 4 mM amino acids; L-Val (open circle), D-Val (closed circle), **(B)** L-Leu (open square) and D-Leu (closed square).

During its growth with branched-chain AAs such as Val and Leu, strain A04V preferred the D-forms for growth rather than the L-forms (Figure 4). The preference for the D-forms of AAs was not observed when we tested other AAs such as Ala, Glu, and Phe as sole carbon and nitrogen sources (Figure S1). The optimum D-Val concentration for growth was 5–6 mM. Adverse effects of high concentrations of DAAs have been reported previously in a variety of bacteria (Teeri and Josselyn, 1953; Bopp, 1965; Champney and Jensen, 1970; Cosloy and McFall, 1973; Yabu and Huempfner, 1974; White, 1979). The growth of strain A04V was also completely inhibited in the presence of >10 mM D-Val. In contrast, high concentrations (< 100 mM) of L-Val did not inhibit the growth of this strain at all. High concentrations of DAAs induce significant morphological changes for *Vibrio cholerae* cells (Lam et al., 2009), but such effect was not observed for strain A04V even when it was incubated with a lethal level of D-Val (10 mM).

**Figure 5:**
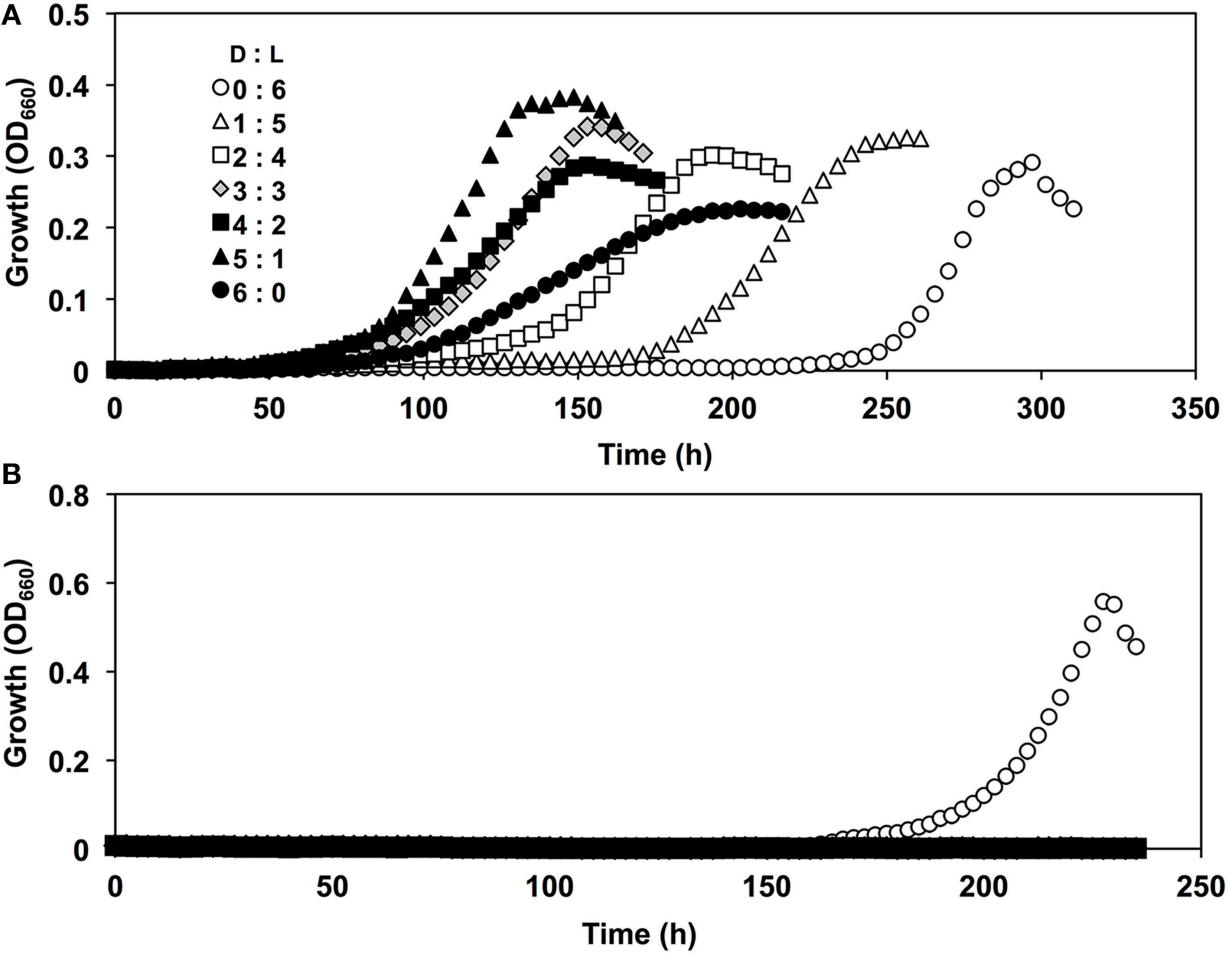
**Growth curves of *Nautella* strains in the presence of D, L-valine**. Strains A04V **(A)** and LMG 24365 **(B)** in the presence of various concentrations of D-Val and L-Val: D:L = 0:6 mM (open circles), D:L = 1:5 mM (open triangles), D:L = 2:4 mM (open squares), D:L = 3:3 mM (light-gray diamonds), D:L = 4:2 mM (filled squares), D:L = 5:1 mM (filled triangles), and D:L = 6:0 mM (filled circles).

Moreover, strain A04V exhibited unique growth behavior in the presence of racemic Val, i.e., a mixture of L- and D-Val (the final total Val concentration was 6 mM), as the sole carbon and nitrogen source (Figure 5A). Growth was robust in the presence of both Val enantiomers, whereas growth was weak with each separate enantiomer of Val. In contrast, the type strain of *N*. *italica* (strain LMG24365), which was isolated from a shallow-sea environment (Vandecandelaere et al., 2009), grew only with L-Val, and not with D-Val or racemic mixtures (Figure 5B). As strain A04V and *N*. *italica* LMG24365 share 100% similarity in terms of its 16S rRNA genes, the capacity for utilizing D-Val as the sole carbon and nitrogen source for growth is not a common feature of this genus.

**Figure 6:**
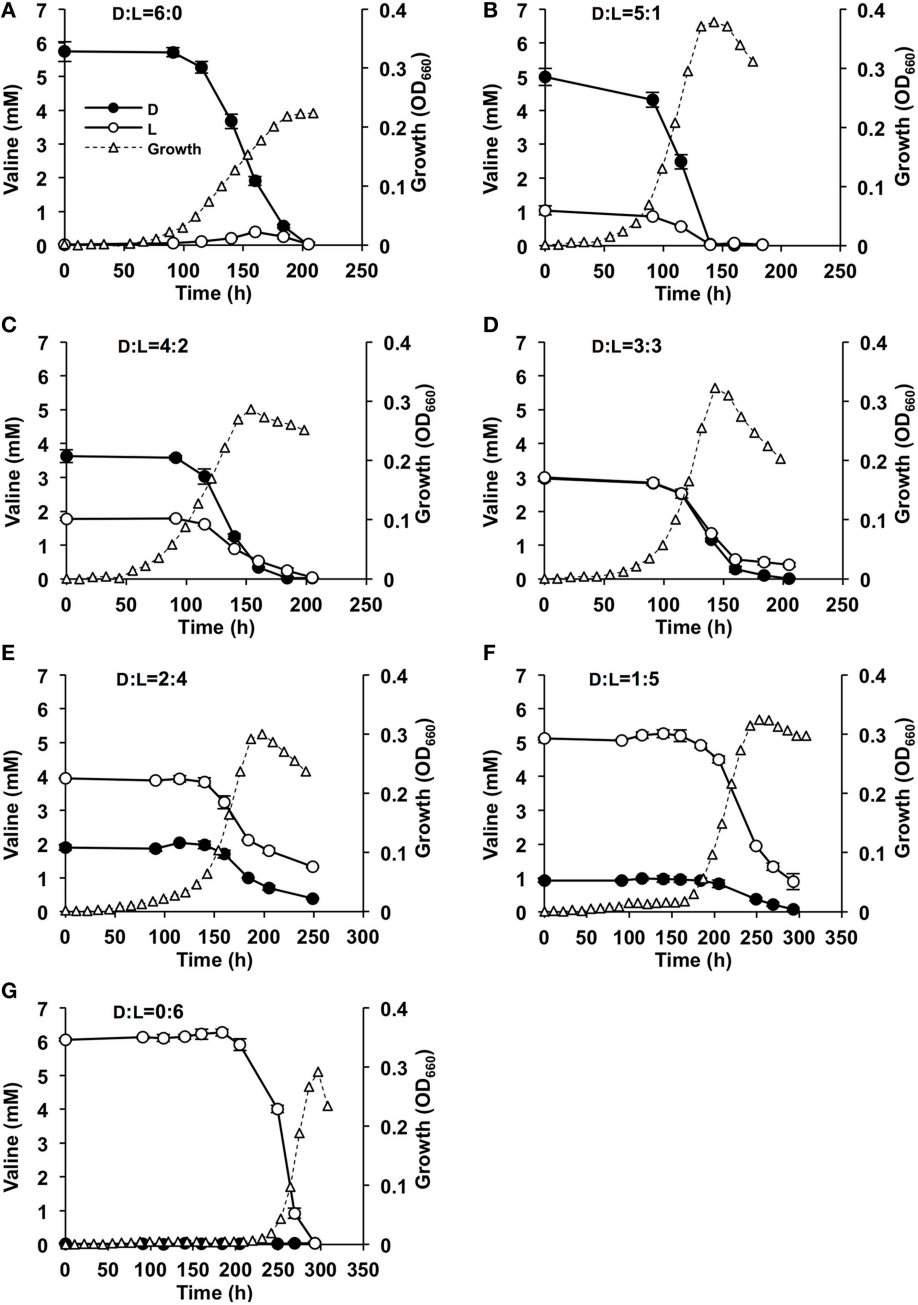
**Effects of valine concentrations in the medium on the growth of ***Nautella*** sp. strain A04V**. The substrates used for cultivation were: 6 mM D-Val **(A)**, 6 mM racemic Val at D:L = 5:1 mM **(B)**, D:L = 4:2 mM **(C)**, D:L = 3:3 mM **(D)**, D:L = 2:4 mM **(E)**, D:L = 1:5 mM **(F)**, and 6 mM L-Val **(G)** as the sole carbon and nitrogen sources. The concentrations of D-Val (filled circles) and L-Val (open circles) were determined by HPLC. The growth of the strain was measured instrumentally by determining the optical density at 660 nm (OD_660_) (open triangles).

The rate of consumption of Val (D-, L-, and D, L-Val) during the growth of strain A04V was determined by HPLC (Figure 6). D-, L-, and D, L-Val were completely depleted from the medium in the early stationary phase. Intriguingly, D-Val was consumed faster than L-Val during cultivation when the L- or D-form was supplemented as sole carbon and nitrogen sources, whereas L-Val and D-Val were consumed at the same rate when racemic mixtures were used (Figure 6).

In a soil microbial assemblage, LAAs were consumed faster than DAA except for alanine (Zhang and Sun, 2014). A lag time was observed to induce racemase for conversion of D-enantiomers to L-enantiomers except for alanine racemases that are synthesized constitutively in most microbes (Zhang and Sun, 2014). The marine isolate A04V was distinctly different. Although it consumed D- and L-alanine at equal rates (Figure S1), the consumption rates of D-enantiomers of branched-chain AAs in strain A04V were faster than L-enantiomers.

### DAA degradation activity of strain A04V and close relatives

**Figure 7:**
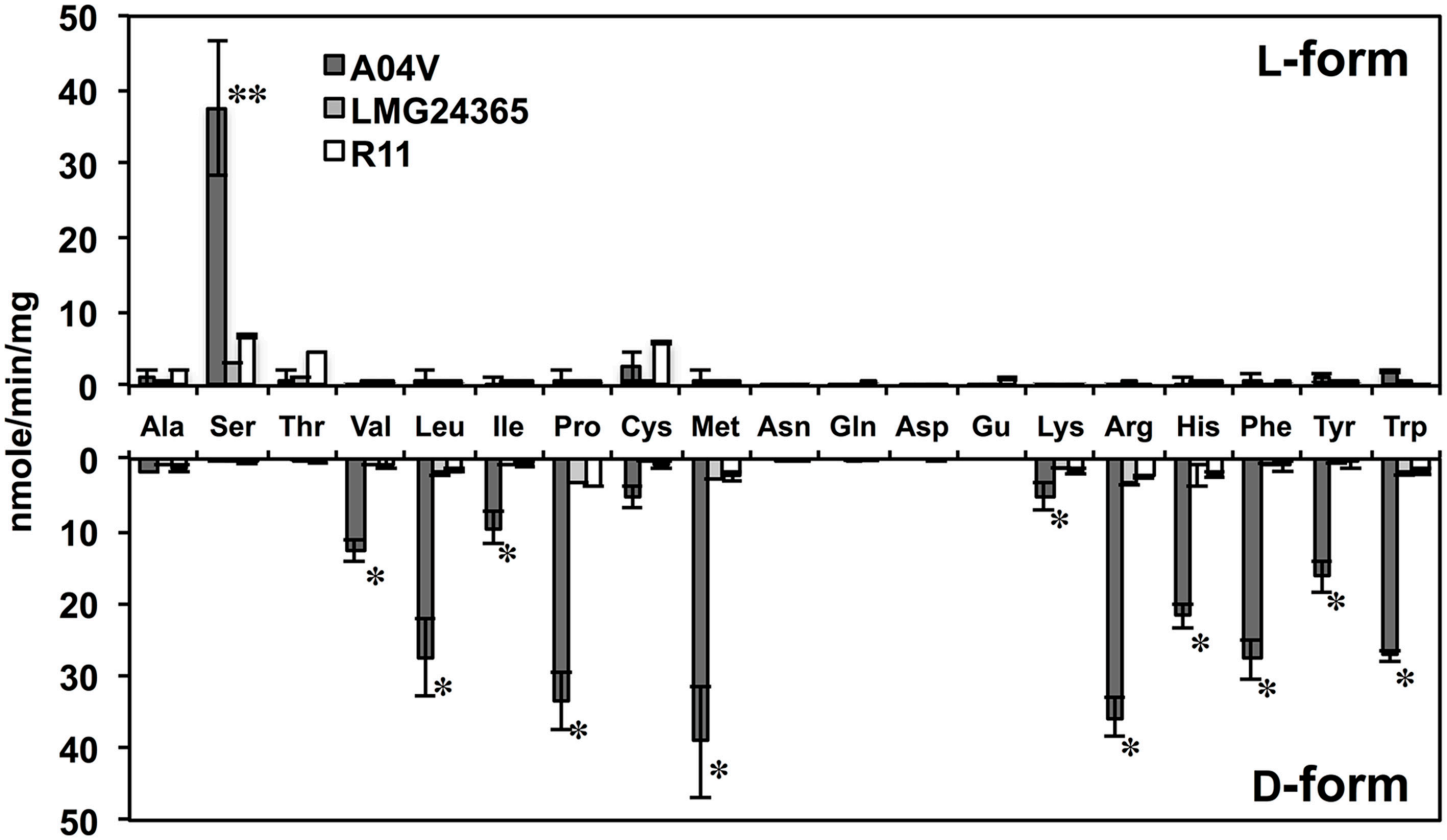
**Deamination of amino acids to α-keto acids catalyzed by resting cells of strains A04V, ***N. italica*** LMG24365, and R11**. Reactions were performed at 30°C for 1 h. Double asterisks (^**^) represent amino acids with L-enantiomers that were preferentially deaminated and the single asterisk (^*^) denotes those that deamination occurred preferentially for D-enantiomers. The data represent the mean ± SD (indicated by vertical bars) based on at least two independent experiments.

To elucidate the biochemical features of strain A04V, we compared the DAA degradation activities of *N*. *italica* strains A04V and LMG24365. The catalytic activities of the resting cells of strain A04V indicated that they could degrade various DAAs (Figure 7). DAA degradation activities were also detected in the resting cells of *N*. *italica* strains LMG24365, but they were extremely low compared with those of strain A04V (Figure 7). Both strains showed similar substrate specificities.

We also determined the kinetic parameters of the DAA degradation activities of these strains. The catalytic activity (*V*_max_) of valine degradation of strain A04V was 20 times higher than that of LMG24365 (20.58 ± 2.88, 1.11 ± 0.02), but both strains showed similar substrate affinity (*K*_m_) (5.68 ± 0.57 for A04V, 8.77 ± 2.0 for LMG24365) (Table S3).

Enzymatic degradation of DAAs did not occur when the disrupted cells of *Nautella* strains, including the cell wall and membrane fraction, were used in the reaction, indicating that the enzymes involved in enantioselective degradation are membrane-bound proteins and/or located in the periplasmic space. Similar results were previously reported for bacterial DAA deaminases (Tsukada, 1966; Olsiewski et al., 1980). These enzymes catalyze deamination of various DAAs, but works especially well for branched-chain, basic, and aromatic DAAs (Tsukada, 1966; Olsiewski et al., 1980; Satomura et al., 2002; Li et al., 2010). The DAA degradation activities of the A04V cells showed very similar substrate specificity to the known DAA deaminases (Figure 7). DAA aminotransferases are also involved in DAA metabolic pathway in several bacteria (Tanizawa et al., 1989; Lee et al., 2006). The DAA aminotransferases catalyze the transamination using the several DAAs as substrates, but they show no or weak activities for branched-chain and aromatic DAAs (Tanizawa et al., 1989; Lee et al., 2006). Accordingly, the DAA degradation activities of the Nautella strains are likely catalyzed by DAA deaminases.

### Genomic analysis of *Nautella* strains

A complete genome sequence is only available for *N*. *italica* strain R11 in this genus (Fernandes et al., 2011), which was isolated as a pathogen causing a bleaching disease of red algae (Case et al., 2011). The strain showed DAA degradation activities similar to strain LMG24365 (Figure 7, Table S3), and grew with L-Val but not D-Val. Thus, we analyzed genome sequences of strains A04V and LMG24365, and performed comparative genome analysis of the three strains.

The genome sequences were analyzed using the Illumina HiSeq 2000 platform to identify the genomic backgrounds of DAA degradation in strain A04V. In total, we obtained 7,101,444 and 7,191,597 reads (90 bp) for strains A04V and LMG24365, respectively (Table S4). After removing low-quality reads, these reads were then mapped onto the genomic sequence of strain R11. The mapped reads shared 87% (6,190,244 out of 7,101,444 reads) of A04V and 90% (6,522,580 out of 7,191,597 reads) of LMG24365 in each of the total reads. The coverage rates were 94.9% (depth: 132.4 ×) and 96% (depth: 137.6 ×), respectively, relative to the reference genome of strain R11. The average GC contents were 59%. The average nucleotide similarities with *N*. *italica* strain R11 were 97% (A04V) and 98% (LMG24365), respectively.

The genome sequences from strain R11 contained six predicted CDSs for putative DAA oxidases (or dehydrogenases) (pfam01266) and four CDSs for putative DAA transferases (pfam01063). CDSs were also identified in the draft genomes of strains A04V and, LMG24365. The shared amino acid identities of the genes among the three strains were 99–100% and only non-conservative amino acid substitutions were observed (Table 1). Furthermore, we found no other candidate genes related to DAA degradation by BLAST and Pfam analyses of the mapped reads from strain A04V.

**Table 1:** **Comparisons of the CDSs encoding known DAA deaminases among ***Nautella*** strains**.

**Pfam**	**Protein ID in strain R11**	**Annotation**	**Shared identity (%)**		**Amino acid substitution**	
			**A04V**	**LMG24365**	**A04V**	**LMG24365**
**DAO (Pfam01266: D-AMINO ACID OXIDASE)**						
	RR11_23	Glycine/D-amino acid oxidase, deaminating	99	100	5	0
	RR11_898	D-Amino acid dehydrogenase	99	100	2	0
	RR11_1993	D-Amino acid dehydrogenase small subunit	99	100	4	0
	RR11_2500	Glycine/D-amino acid oxidase	99	100	4	0
	RR11_2832	Glycerol-3-phosphate dehydrogenase	99	100	2	0
	RR11_2957	Glycine/D-amino acid oxidase	99	100	3	0
**AMINOTRAN_4 (Pfam01063: D-AMINO ACID TRANSFERASES)**						
	RR11_862	D-Alanine aminotransferase	100	100	0	0
	RR11_1631	Branched-chain amino acid aminotransferase	100	100	0	0
	RR11_2182	Branched-chain amino acid aminotransferase	99	99	2	2
	RR11_3119	Aminotransferase, class IV	99	100	1	0

*The accession numbers of the sequences are shown in Table S2*.

The unmapped reads for A04V and LMG24365 were assembled into 2,587 (N50 contig size: 613) and 716 (N50 contig size: 1,425) contigs, respectively. Analysis of the contig sequences of A04V detected 696 candidate peptide sequences, which contained 300 sequences similar to those of LMG24365. As a result, we found no candidate genes encoding DAA degradation enzymes among these sequences. According to the comparative genome analysis, we observed little difference between the genes involved with the known DAA metabolism processes in the three *Nautella* strains. Thus, we suggest that a few differences in terms of amino acid substitutions might explain the differences in DAA utilization among the three strains. However, according to our analysis, the amino acid substitutions in the six genes that encoded the putative DAA deaminase of strain A04V (shown in Table 1) had no significant relationships with the bacterial habitats based on analyses of the corresponding gene sequences in bacterial genome databases (including the sequences of the environmental sample).

Moreover, the six genes identified by the genomic analysis (shown in Table 1) as encoding putative DAA deaminases were cloned and expressed in *E. coli*. However, no significant activities were detected in the recombinant *E. coli* cells or the cell extracts (unpublished data), which suggests that the gene products require other components for activation such as membrane-associated molecules.

## Conclusion

Microbiological and geochemical studies suggested the microbial production and consumption of DAAs in pelagic water ecosystems (Perez et al., 2003; Teira et al., 2006; Calleja et al., 2013; Zhang et al., 2016). In this geochemical cycle, importance of marine Alphaproteobacteria in the production of DAAs has already been pointed out (Pedersen et al., 2001; Lomstein et al., 2006; Kaiser and Benner, 2008; Kubota et al., 2016), however, phylogenetic and physiological features of DAA-utilizing marine bacteria are poorly investigated yet.

In this study, various DAA-utilizing bacteria were successfully isolated from deep-sea environments, and their catalytic activities with L- and D-AAs were evaluated using resting cells. Especially, the resting cells of the several alphaproteobacterial isolates showed significantly higher DAA degradation activities than those of other isolates. The strain A04V exhibited robust growth on medium supplemented with D-Val, whereas its growth was poor on minimal medium supplemented with L-Val as a sole carbon and nitrogen source. In contrast, other *Nautella* strains (strain R11 and LMG24365) isolated from a shallow-sea environment grew with L-Val but not D-Val. The catalytic activity of the DAA degradation in the strain A04V was 20 times greater than that of the other two *Nautella* strains. However, there are no significant differences in the gene sets involved with the DAA metabolisms among the three *Nautella* strains. These results suggest that minor genetic variance in the gene sets involved DAA metabolisms and/or gene regulation influence the variance of DAA utilization among the strains. Therefore, combination of reverse genetics and biochemical analyses is necessary to understand the metabolism and physiological role of DAAs in the marine DAA-utilizing bacteria.

## Author contributions

TaK carried out conception and design of the study, analysis and interpretation of data, collection and assembly of data, drafting of the article, and critical revision of the article for important intellectual content. ToK, SD conceived of the study, and participated in its design and coordination and helped to draft the manuscript. TN, FM interpreted of data of the molecular genetic studies for the work and helped to drafted the manuscript. All authors read and approved the final manuscript.

### Conflict of interest statement

The authors declare that the research was conducted in the absence of any commercial or financial relationships that could be construed as a potential conflict of interest.

## Abbreviations

DAA: D-amino acid
LAA: L-amino acid
MBTH: 3-methyl-2-benzothiazolinone hydrazine
TCA: Trichloroacetic acid
DOM: dissolved organic matter.
