# Supplementary material for: Enantioselective Utilization of D-Amino Acids by Deep-Sea Microorganisms
Source: Front Microbiol. 2016 Apr 19;7:511. doi: 10.3389/fmicb.2016.00511 (PMC4836201; doi:10.3389/fmicb.2016.00511)
Supplement: Supplementary file 1 [file Table1.DOCX]

**Table S1. Descriptions of the sampling sites and dates**

| Cruise No. | Dive No. | Location (Lat., Long.) | Depth (m) | Date |
| --- | --- | --- | --- | --- |
| Cruise NT01-11 | 2K#1325 | 35°0.076N, 139°13.510E | 1168 | December 03, 2001 |
|  | 2K#1326 | 35°0.245N, 139°13.481E | 1164 | December 04, 2001 |
|  | 2K#1327 | 35°0.076N, 139°13.465E | 1163 | December 05, 2001 |
| NT04-06 | HPD#303 | 35°0.771N, 139°21.635E | 1453 | June 12, 2004 |
|  | HPD#304 | 35°0.813N, 139°21.633E | 1453 | June 13, 2004 |
|  | HPD#305 | 35°0.069N, 139°13.444E | 1157 | June 14, 2004 |
| YK05-15 | 6K-#913 | 35°0.846N, 139°13.601E | 1120 | December 07, 2005 |
|  | 6K-#914 | 35°0.061N, 139°13.430E | 1153 | December 08, 2005 |
|  | 6K-#915 | 35°5.901N, 139°20.643E | 1168 | December 09, 2005 |
|  | 6K-#916 | 35°0.951N, 139°13.389E | 853 | December 10, 2005 |
|  | 6K-#917 | 35°0.051N, 139°13.418E | 1152 | December 11, 2005 |
| NT06-01 | HPD#514 | 35°4.996N, 139°13.013E | 927 | January 20, 2006 |
| NT06-17 | HPD#594 | 35°4.993N, 139°13.024E | 926 | August 21, 2006 |
|  | HPD#595 | 35°4.990N, 139°13.021E | 926 | August 22, 2006 |
| YK07-05 | 6K-#1008 | 35°0.9441N, 139°13.3181E | 852 | April 07, 2007 |
|  | 6K-#1009 | 35°0.9421N, 139°13.3858E | 903 | April 09, 2007 |
| NT08-24 | HPD#921 | 35°4.988N, 139°13.018E | 928 | December 08, 2008 |
|  | HPD#922 | 35°0.957N, 139°13.333E | 856 | December 09, 2008 |
|  | HPD#923 | 35°0.954N, 139°13.336E | 856 | December 10, 2008 |
|  | HPD#924 | 35°0.937N, 139°13.376E | 912 | December 10, 2008 |
