# Supplementary material for: Enantioselective Utilization of D-Amino Acids by Deep-Sea Microorganisms
Source: Front Microbiol. 2016 Apr 19;7:511. doi: 10.3389/fmicb.2016.00511 (PMC4836201; doi:10.3389/fmicb.2016.00511)
Supplement: Supplementary file 2 [file Table2.DOCX]

**Table S2. The accession numbers of the sequences in this study**

| Accession No. | Sequence | Strain ID |
| --- | --- | --- |
| LC095000 | partial 16S ribosomal RNA | *Nautella* sp. B30Va |
| LC095004 | partial 16S ribosomal RNA | *Nautella* sp. A04F |
| LC094989 | partial 16S ribosomal RNA | *Nautella* sp. A06V |
| LC094988 | partial 16S ribosomal RNA | *Nautella* sp. A04V |
| LC095007 | partial 16S ribosomal RNA | *Thalassobius* sp. A38Fa |
| LC095013 | partial 16S ribosomal RNA | *Thalassobius* sp. B54Fa |
| LC095015 | partial 16S ribosomal RNA | *Ruegeria* sp. B28Fb |
| LC094997 | partial 16S ribosomal RNA | *Ruegeria* sp. B04V |
| LC094996 | partial 16S ribosomal RNA | *Ruegeria* sp. B18F |
| LC094995 | partial 16S ribosomal RNA | *Ruegeria* sp. B01Va |
| LC095008 | partial 16S ribosomal RNA | *Tropicibacter* sp. B01Fbb |
| LC095012 | partial 16S ribosomal RNA | *Tropicibacter* sp. B38Fa |
| LC094990 | partial 16S ribosomal RNA | *Paracoccus* sp. A18Va |
| LC094992 | partial 16S ribosomal RNA | *Paracoccus* sp. A30V |
| LC095001 | partial 16S ribosomal RNA | *Labrenzia* sp. B30Vb |
| LC095009 | partial 16S ribosomal RNA | *Rhizobium* sp. B22Fa |
| LC094991 | partial 16S ribosomal RNA | *Rhizobium* sp. A22V |
| LC095002 | partial 16S ribosomal RNA | *Marinobacter* sp. B30Vc |
| LC095011 | partial 16S ribosomal RNA | *Marinobacter* sp. B30F |
| LC095006 | partial 16S ribosomal RNA | *Pseudomonas* sp. A11Fb |
| LC094999 | partial 16S ribosomal RNA | *Rheinheimera* sp. B18Va |
| LC095010 | partial 16S ribosomal RNA | *Marinomonas* sp. B22Fb |
| LC095005 | partial 16S ribosomal RNA | *Bacillus* sp. A06F |
| LC095003 | partial 16S ribosomal RNA | *Bacillus* sp. B53V |
| LC094993 | partial 16S ribosomal RNA | *Bacillus* sp. A32Vb |
| LC094998 | partial 16S ribosomal RNA | *Bacillus* sp. B17Va |
| LC094994 | partial 16S ribosomal RNA | *Bacillus* sp. A46V |
| LC095014 | partial 16S ribosomal RNA | *Jeotgalibacillus* sp. B54Fb |
| PRJDB4276 | draft genome sequence | *Nautella* sp. A04V |
| PRJDB4510 | draft genome sequence | *Nautella* *italica* LMG24365 |
| LC124156 | glycine/D-amino acid oxidase | *Nautella* sp. A04V |
| LC124157 | glycine/D-amino acid oxidase | *Nautella* *italica* LMG24365 |
| LC124158 | D-amino acid dehydrogenase | *Nautella* sp. A04V |
| LC124159 | D-amino acid dehydrogenase | *Nautella* *italica* LMG24365 |
| LC124160 | D-amino acid dehydrogenase small subunit | *Nautella* sp. A04V |
| LC124161 | D-amino acid dehydrogenase small subunit | *Nautella* *italica* LMG24365 |
| LC124162 | glycine/D-amino acid oxidase | *Nautella* sp. A04V |
| LC124163 | glycine/D-amino acid oxidase | *Nautella* *italica* LMG24365 |
| LC124164 | glycerol-3-phosphate dehydrogenase | *Nautella* sp. A04V |
| LC124165 | glycerol-3-phosphate dehydrogenase | *Nautella* *italica* LMG24365 |
| LC124166 | glycine/D-amino acid oxidase | *Nautella* sp. A04V |
| LC124167 | glycine/D-amino acid oxidase | *Nautella* *italica* LMG24365 |
| LC124168 | D-alanine aminotransferase | *Nautella* sp. A04V |
| LC124169 | D-alanine aminotransferase | *Nautella* *italica* LMG24365 |
| LC124170 | branched-chain amino acid aminotransferase | *Nautella* sp. A04V |
| LC124171 | branched-chain amino acid aminotransferase | *Nautella* *italica* LMG24365 |
| LC124172 | branched-chain amino acid aminotransferase | *Nautella* sp. A04V |
| LC124173 | branched-chain amino acid aminotransferase | *Nautella* *italica* LMG24365 |
| LC124174 | aminotransferase class IV | *Nautella* sp. A04V |
| LC124175 | aminotransferase class IV | *Nautella* *italica* LMG24365 |
