# Supplementary material for: Enantioselective Utilization of D-Amino Acids by Deep-Sea Microorganisms
Source: Front Microbiol. 2016 Apr 19;7:511. doi: 10.3389/fmicb.2016.00511 (PMC4836201; doi:10.3389/fmicb.2016.00511)
Supplement: Supplementary file 3 [file Table3.docx]

**Table S3. Kinetic properties of D-valine degradation by resting cells of *Nautella* strains.**

|  | *V*_max_ | *K*_m_ |
| --- | --- | --- |
| Strain ID | [nmole min^-1^ mg^-1^] | [mM] |
| A04V | 20.58 ± 2.88 | 5.68 ± 0.57 |
| *N. italica* LMG24365 | 1.11 ± 0.02 | 8.77 ± 2.00 |
| *N. italica* R11 | 1.39 ± 0.05 | 4.28 ± 0.76 |
| The data sets are mean ± SD of duplicate or triplicate sets. | | |
