# Supplementary material for: Enantioselective Utilization of D-Amino Acids by Deep-Sea Microorganisms
Source: Front Microbiol. 2016 Apr 19;7:511. doi: 10.3389/fmicb.2016.00511 (PMC4836201; doi:10.3389/fmicb.2016.00511)
Supplement: Supplementary file 4 [file Table4.DOCX]

**Table S4. Genome features of *Nautella* strains**

|  | | Strain | |
| --- | --- | --- | --- |
|  |  | A04V | LMG24365^T^ |
| Genome analysis | | | |
|  | Similarity with strain R11 (%) | 97 | 98 |
|  | GC content (mol%) | 59 | 59 |
| Mapping with strain R11 | | | |
|  | Coverage (%) | 95 | 96 |
|  | Depth | 132.4× | 137.6× |
