# Supplementary material for: Enantioselective Utilization of D-Amino Acids by Deep-Sea Microorganisms
Source: Front Microbiol. 2016 Apr 19;7:511. doi: 10.3389/fmicb.2016.00511 (PMC4836201; doi:10.3389/fmicb.2016.00511)
Supplement: Supplementary file 5 [file DataSheet1.DOCX]

**Legends of Figures**

**Figure S1.** **Growth curves of *Nautella* sp. strain A04V.** In the presence of several L- and D-amino acids at 4 mM; L-Ala (open circle), D-Ala (closed circle) L-Glu (open triangle), D-Glu (closed triangle), L-Phe (open diamond) and D-Phe (closed diamond).
