# Supplementary figures and images for: Enantioselective Utilization of D-Amino Acids by Deep-Sea Microorganisms
Source: Front Microbiol. 2016 Apr 19;7:511. doi: 10.3389/fmicb.2016.00511 (PMC4836201; doi:10.3389/fmicb.2016.00511)

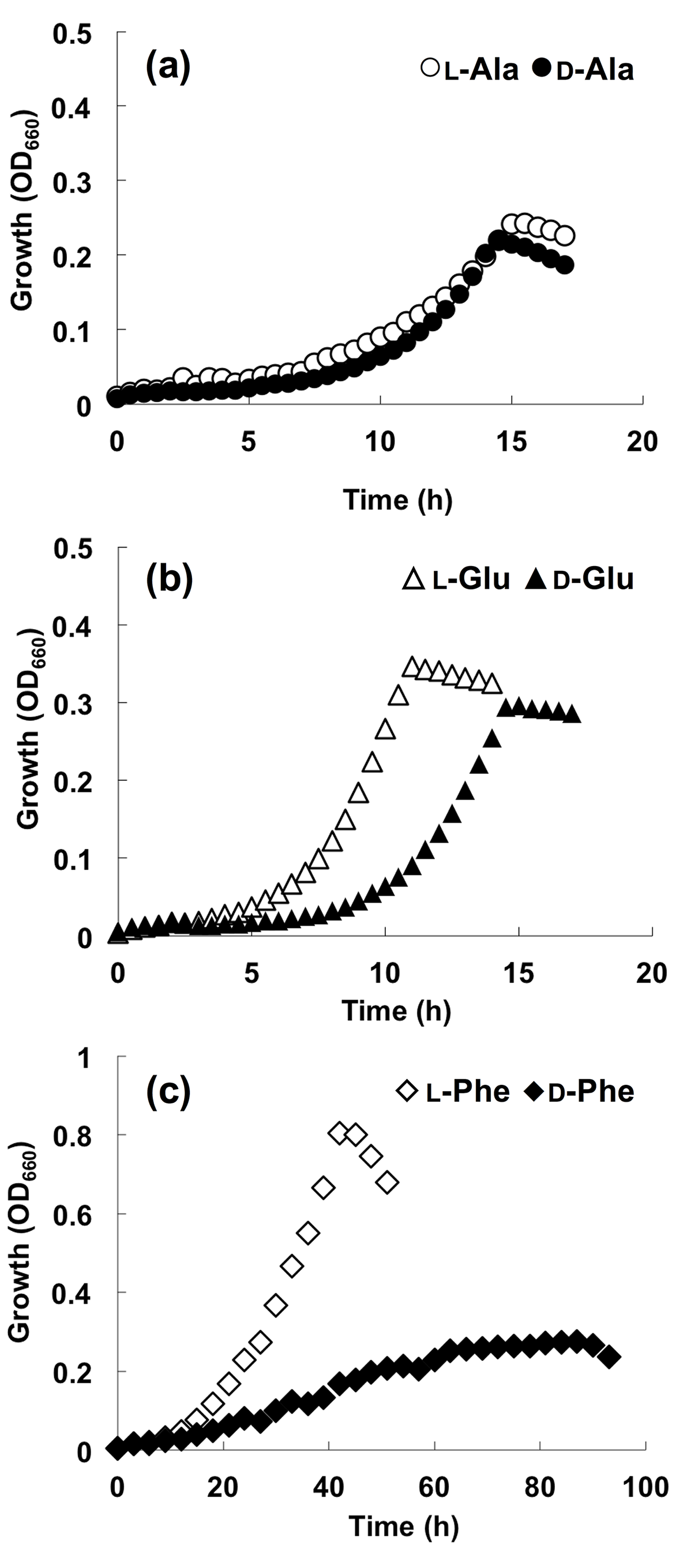

Supplement: Supplementary file 6 [file Image1.tif]
